# Diagnosis and Management of a Hypersensitivity Reaction to Titanium-Containing Surgical Clips: A Case Report

**DOI:** 10.7759/cureus.34929

**Published:** 2023-02-13

**Authors:** Darren N Ramcharan, Kayla L Alaimo, Frederick Tiesenga

**Affiliations:** 1 Medicine, Saint James School of Medicine, Park Ridge, USA; 2 General Surgery, West Suburban Medical Center, Chicago, USA

**Keywords:** metal-ltt, melisa, case report, low-dose naltrexone, patch testing, titanium allergy, hypersensitivity

## Abstract

Although titanium allergies are not commonly diagnosed, they can present with a variety of conditions years after the implantation of titanium-containing medical devices. Furthermore, there are few options to effectively manage the long-term outcomes of these conditions. We present the case of a 41-year-old female who experienced neck swelling, pain, and difficulty swallowing 16 years after a right thyroid lobectomy for benign follicular adenoma, requiring the implantation of titanium-containing surgical clips in her neck. This was accompanied by an extensive symptomatic history, and the patient showed mild reactivity to nickel and titanium on a metal lymphocyte transformation test analysis. X-ray and computed tomography of the neck later confirmed the location of 18 surgical clips. The patient was diagnosed with a chronic immune disease including immune complex disease and mast cell activation-related symptoms. Symptoms were managed with low-dose naltrexone until the surgical clips were removed. Further research is needed to identify more accurate testing methods to diagnose titanium hypersensitivity. Alternative treatment methods should be explored to reduce disease burden and complications related to titanium-containing implants.

## Introduction

Nickel, cobalt, chromium, and titanium are just some of the many metallic elements used widely in dental and surgical implants. While nickel has a well-documented history of causing hypersensitivity reactions, with an estimated prevalence of 17% in women and 3% in men in some studies, titanium allergies are less frequently diagnosed [1]. Titanium alloys are desirable for implantable devices due to their biocompatibility and resistance to corrosion within body tissues. Implantation of titanium-containing devices increases the metal concentration and exposure to bodily fluids or tissues. This increases the likelihood of allergic or hypersensitivity reactions [2]. Metallic ions and particles released into the surrounding environment may generate molecules capable of triggering immune reactions. There are multiple mechanisms by which metallic ions are believed to cause hypersensitivity reactions to metal alloys. Proposed mechanisms include sensitization of T cells to hapten-like molecules or phagocytosis and activation of macrophages. The activation of immune cells may lead to the release of pro-inflammatory cytokines and stimulation of B cells to produce IgE and IgG antibodies to metal-containing molecules [3]. Reactions to metallic ions tend to have systemic effects and manifest as a broad range of signs and symptoms between individual cases, making it difficult to diagnose cases purely on clinical presentation.

Prior notable cases of suspected metal reactions encountered include a 61-year-old female who had complaints of nonspecific symptoms following surgical clip placement during cholecystectomy. This patient had a history of a localized allergic reaction to titanium plates after ankle surgery. A review of medical records showed her memory lymphocyte immunostimulation assay (MELISA) had equivocal results for titanium allergy [4]. Also encountered was a 28-year-old female who presented in 2016 with a variety of symptoms 11 months after cholecystectomy with surgical clip placement. This patient had a history of delayed hypersensitivity to nickel and other metal allergies with documented positive titanium skin patch test but equivocal MELISA results. Laparoscopic removal of the clips led to the resolution of symptoms in one month [5]. Although surgical removal of metallic foreign bodies is the typical intervention for these conditions, novel drug therapies such as low-dose naltrexone (LDN) may provide the benefit of symptomatic relief until surgical intervention due to their immunomodulatory effects [6]. In this report, we present a case of a suspected titanium hypersensitivity reaction managed with LDN and the removal of surgical clips.

## Case presentation

Chief complaint

**Figure 1:**
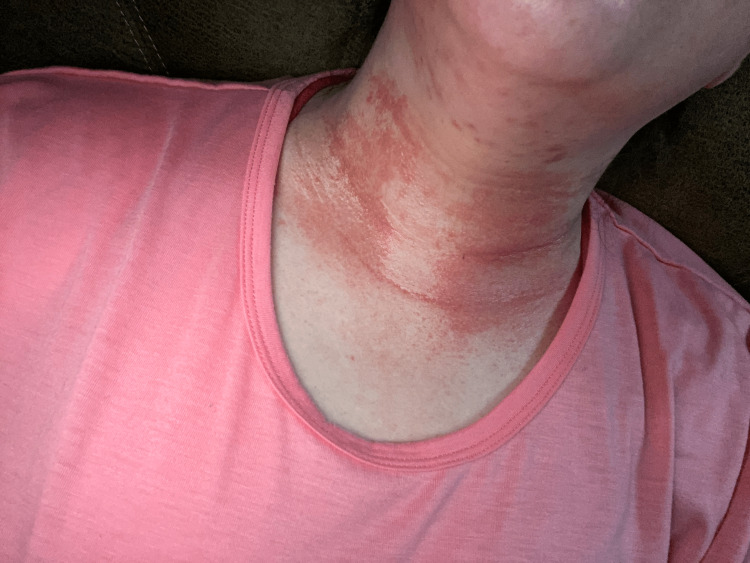
Swelling and rash visualized on the anterior neck.

A 41-year-old female presented to her primary care physician (PCP) in August 2021 complaining of worsening neck pain, swelling, and difficulty swallowing at the site of a previous partial thyroidectomy (Figure 1). She also reported having associated intermittent nausea and tenesmus, episodes of presyncope, and full-body tremors triggered by crouching, leaning, sitting, or standing upright for extended periods at the time of presentation.

History of present illness

The patient contacted the surgery clinic after her PCP referred her for an X-ray and a three-dimensional computed tomography (3D CT) scan in October 2021 due to suspicion of delayed hypersensitivity reaction to surgical clip implantation in her neck. Imaging revealed 18 surgical clips that were implanted in her neck during a right thyroid lobectomy for the removal of a benign follicular adenoma in 2005.

The patient reported a majority of her symptoms occurred in 2015 or later that were described by her PCP as a “mono-like” syndrome, although she had negative mononucleosis antibody titers when evaluated. She first experienced extreme fatigue and general malaise associated with difficulty ambulating and speaking, as well as general left-sided abdominal pain. Additional symptoms experienced by the patient over the last 10 years include but are not limited to memory loss, difficulty concentrating, severe shortness of breath, hot and cold flashes, metallic taste in the mouth, halitosis, change in body odor, abdominal bloating, decreased libido, joint and body aches, muscle pain and tightness, bruxism, insomnia, dizziness, sudden-onset nausea, diarrhea, constipation, blurred vision, hair loss, skin rash, dermatitis, tachycardia, palpitations, weight gain, mood swings, coarse facial hair growth, increasing frequency of asthma exacerbations, paresthesia of extremities, migraines, acne, anovulation, lymphadenopathy with associated pain, swelling of hands and feet, mild fevers, sore throat, cough, laryngitis without a sore throat, thyroid swelling, tinnitus, and fecal incontinence. She reported an episode of syncope in 2019 and described brain fog and episodic fasciculations throughout 2020. At the time of the encounter, the patient reported her current symptomatology consistent with her chief complaint while awaiting surgical removal of the implanted clips.

Past medical history

The patient’s past medical history consisted of type 2 diabetes, mixed hyperlipidemia, nonalcoholic steatohepatitis, pancreatitis, splenomegaly, and autonomic neuropathy. The patient was also diagnosed with atypical depression and was prescribed mirtazapine which temporarily relieved her insomnia. However, when the depression symptoms returned, mirtazapine was discontinued. The patient was currently on 15 mg of melatonin to aid with sleep. The patient’s past surgical history included bilateral tubal ligation with Filshie clip placement in 2010 as well as laparoscopic cholecystectomy with surgical clip placement in 2005. The patient’s social history was insignificant, with no smoking and occasional alcohol consumption. The patient had no known history of allergic reactions or allergies to metals or other substances.

Investigations

Laboratory Investigations

The patient had positive laboratory results for inflammation between 2016 and 2021 (Table 1). Further testing revealed negative results for markers of autoimmune disease and inflammation between 2016 and 2019. Metal lymphocyte transformation test (metal-LTT) performed in 2021 revealed mild reactivity to nickel and titanium metals. Repeat examination for markers of autoimmune disease and inflammation remained within normal limits and not significant in 2022.

**Table 1:** Significant laboratory findings. SBP = systolic blood pressure; DBP = diastolic blood pressure; CRP = C-reactive protein; LSI = lymphocyte stimulation index

	Patient value	Normal range
CRP	0.5 mg/dL	<0.3 mg/dL
Eosinophils	4.4%	1–4%
Blood pressure (SBP)	78–133 mmHg	<120 mmHg
Blood pressure (DBP)	53–114 mmHg	<80 mmHg
LSI (titanium alloy)	2.1	<2.0
LSI (nickel)	2.0	<2.0

Imaging

**Figure 2:**
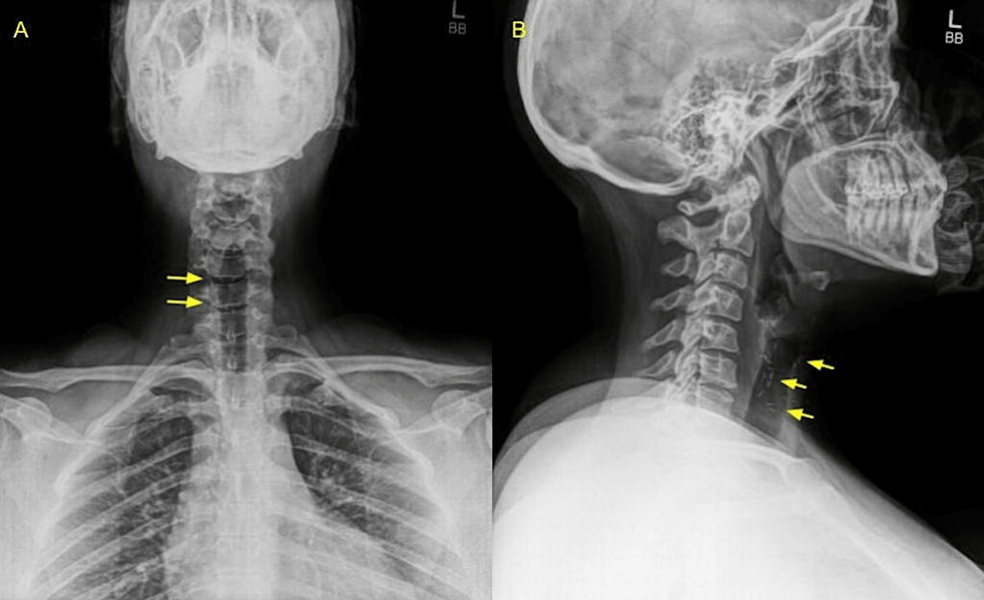
Cervical X-ray with frontal (A) and lateral (B) views of the neck showing surgical clips (yellow arrows).

**Figure 3:**
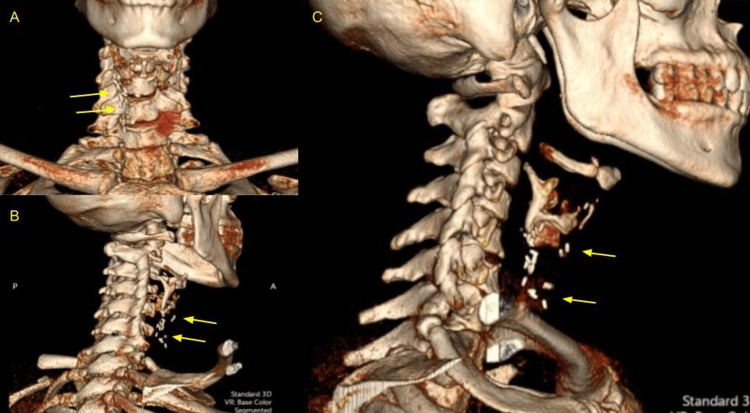
3D CT scan of the neck with frontal (A), posterolateral (B), and lateral (C) views showing surgical clips (yellow arrows).

CT scan of the abdomen and pelvis performed with oral contrast in 2011 confirmed metal surgical clips in the gallbladder fossa and bilateral tubal ligation with Filshie clips. Cervical X-rays (Figure 2) and a CT scan of the neck (Figure 3) performed in October 2021 showed 18 surgical clips in the midline with mild-to-moderate degenerative changes of the cervical spine.

Preoperative diagnosis

Metallic surgical clips (foreign bodies) implanted in the right thyroid bed/parapharyngeal space causing a hypersensitivity reaction resulting in immune complex disease and mast cell activation-related symptoms.

Treatment

**Figure 4:**
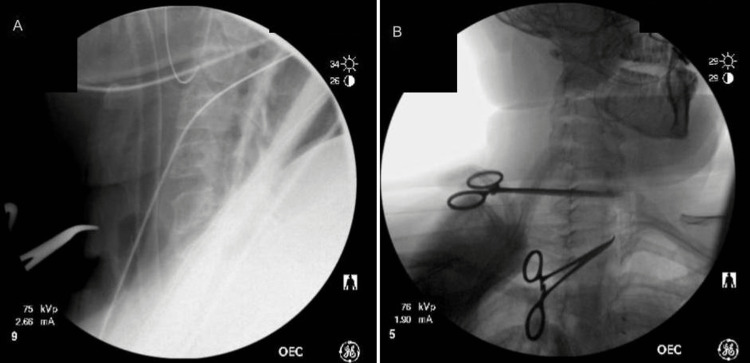
Intraoperative X-ray with lateral (A) and frontal (B) views showing empty right parapharyngeal space.

**Figure 5:**
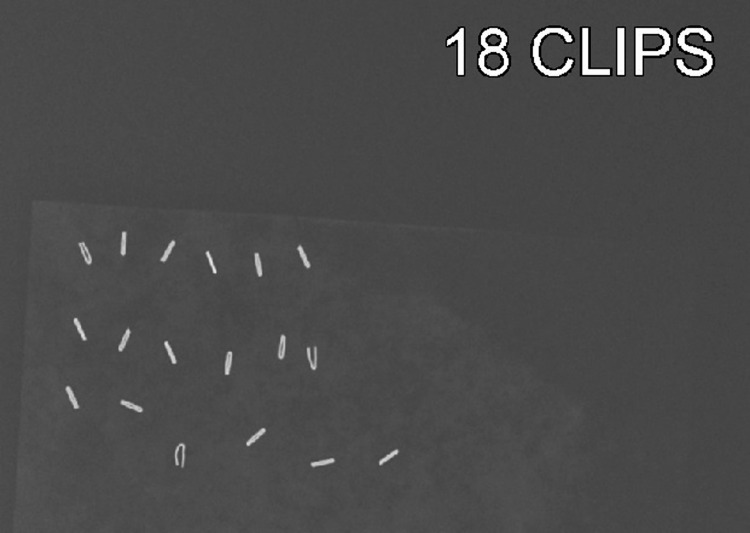
X-ray of 18 surgical clips removed during the procedure.

The patient was prescribed 0.1 mg fludrocortisone after being diagnosed with autonomic neuropathy in 2021 which helped regulate her blood pressure and decreased thyroid pain. After consultation with a rheumatologist in July 2022, the patient was started on LDN therapy of 1.5 mg twice daily to minimize immune activation prior to surgery. This led to an improvement of most autonomic symptoms (dizziness, paresthesia, presyncope, etc.), as well as neck swelling. Removal of 18 surgical clips from the previous right thyroid bed/paratracheal space was performed in December 2022 with one clip remaining due to the inability to properly identify it on X-ray/CT scan prior to the procedure (Figures 4, 5).

Postoperative diagnosis

Hypersensitivity reaction to surgical clips causing suspected immune-related disease inclusive of immune complex disease and mast cell activation-related symptoms.

Outcome/Progress

**Figure 6:**
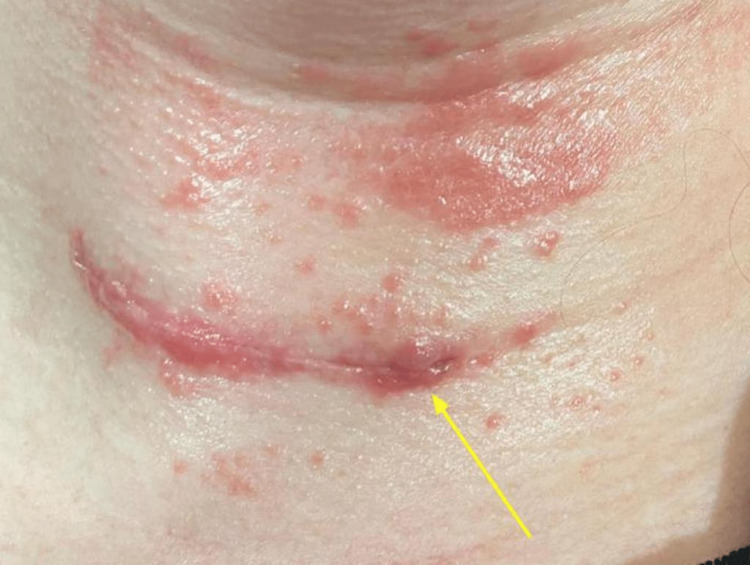
Incisional site with seroma (yellow arrow).

The patient reported resolution of most symptoms within one week of surgical clip removal, complicated by a self-resolving seroma at the incisional site (Figure 6). At the two-month follow-up, the patient reported the appearance of pruritic rashes at the surgical site, which were easily managed with over-the-counter antihistamines. The patient has resumed LDN therapy taking 3 mg daily to minimize hypersensitivity reactions to future metal exposure (diet, environmental).

## Discussion

The challenges presented in patients with complex presentations of hypersensitivity reactions to metallic ions include a lack of availability of validated tools for a reliable diagnosis of these diseases and management of symptoms prior to definitive surgical intervention.

A possible reason for the inaccuracy of current testing methods may be the impurity or variability in the composition of surgically implanted metal devices. Different metallic ions can provide a multitude of immunogenic molecules that induce allergic reactions [7]. Currently, patch testing is the most common method for diagnosing metal allergies, especially in orthopedic procedures, whereby a series of small amounts of metallic compounds are placed in patches on the skin and observed for inflammatory changes. In patients with known metal allergies who have metal-containing implants, nickel has been documented to show strong reactions to patch testing while other metals such as titanium and aluminum powder show no reaction to testing. These inconsistencies may be due to differences in metal salt used in patches, concentrations of compounds, and timings of readings [8]. Prior studies report a sensitivity of about 75% using traditional patch testing for type IV hypersensitivity reactions which may lead us to consider using alternative metal solutions such as titanium sulfate or titanium chloride. These solutions may produce more reliable reagents than titanium oxide, although further studies would need to be conducted. Metal-LTT, as used in this patient’s case, may produce false-positive results by detecting non-relevant lymphocyte proliferation in nonsensitized patients. Therefore, it does not provide reliable, specific, or sensitive results compared to MELISA when diagnosing metal allergies [9]. This suggests that MELISA is the most favorable diagnostic test to use in patients with or without suspected metal allergies or those being considered for metal-containing implants. Interleukin-17 (IL-17) and interleukin-22 (IL-22) may provide a novel diagnostic testing method for metal hypersensitivities as IL-17 has been found to be increased in patients with positive patch testing and IL-22 is implicated in inflammatory reaction of dermatitis to other metals such as nickel. Further studies are needed to determine the reliability and accuracy of these biomarkers in patients with undiagnosed metal allergies [2].

Nonsurgical management of symptoms in patients experiencing metal hypersensitivity reactions has traditionally been with the use of corticosteroids to reduce inflammation but LDN is emerging as an alternative to reduce inflammation without as much concern for harmful side effects. LDN is thought to reduce inflammation by antagonizing mu-opioid receptors paradoxically increasing endogenous endorphin production. Endorphins modulate the function of T-regulatory cells which reduces the production of proinflammatory cytokines and immunoglobulins by T and B cells, respectively. This therapy has been shown to be an effective long-term alternative to surgery or traditional medications in a variety of autoimmune and inflammatory diseases related to immune cell activation, which may allow improved function for patients [10].

## Conclusions

Our approach to patients with suspected metal allergies should consider both the patient’s exposure to metal implants and their history of symptoms. This, however, proves to be challenging when considering the variability of clinical presentations of metal hypersensitivity reactions. Clinical suspicion of these conditions should be able to further guide when to utilize available diagnostic tools. The need to develop more reliable and accurate testing for diagnosis is an important concern in clinical practice with the increasing prevalence of metal allergies, specifically in the fields of dentistry and surgery.

When managing symptoms of patients with confirmed or suspected metal hypersensitivity reactions and related conditions, physicians should consider a multidisciplinary approach utilizing both medical and surgical interventions when appropriate to improve the overall health and outcome for these patients.
